# Association of bone mineral density and fat fraction with magnetic susceptibility in inflamed trabecular bone

**DOI:** 10.1002/mrm.27634

**Published:** 2019-01-07

**Authors:** Timothy J.P. Bray, Anita Karsa, Alan Bainbridge, Naomi Sakai, Shonit Punwani, Margaret A. Hall‐Craggs, Karin Shmueli

**Affiliations:** ^1^ Centre for Medical Imaging University College London United Kingdom; ^2^ Arthritis Research UK Centre for Adolescent Rheumatology University College London United Kingdom; ^3^ Department of Medical Physics and Biomedical Engineering University College London United Kingdom; ^4^ Department of Medical Physics University College London Hospitals United Kingdom

**Keywords:** bone marrow, bone mineral density, inflammation, magnetic susceptibility, proton density fat fraction, QSM, spondyloarthritis

## Abstract

**Purpose:**

To evaluate the relationship between bone mineral density (BMD) and magnetic susceptibility, and between proton density fat fraction and susceptibility, in inflamed trabecular bone.

**Methods:**

Two different phantoms modeling the fat fraction (FF) and BMD values of healthy bone marrow and disease states were scanned using a multiecho gradient echo acquisition at 3T. After correction for fat‐water chemical shift, susceptibility mapping was performed, and susceptibility measurements were compared with BMD and FF values using linear regression. Patients with spondyloarthritis were scanned using the same protocol, and susceptibility values were calculated in areas of inflamed bone (edema) and fat metaplasia, both before and after accounting for the contribution of fat to the total susceptibility.

**Results:**

Susceptibility values in the phantoms were accurately described by a 2D linear function, with a negative correlation between BMD and susceptibility and a positive correlation between FF and susceptibility (adjusted R^2^ = 0.77; *P* = 3·10^−5^). In patients, significant differences in susceptibility were observed between fat metaplasia and normal marrow, but these differences were eliminated by removing the fat contribution to the total susceptibility.

**Conclusions:**

BMD and proton density fat fraction both influence the total susceptibility of bone marrow and failure to account for the fat contribution could lead to errors in BMD quantification. We propose a method for removing the fat contribution from the total susceptibility, based on the observed linear relationship between susceptibility and FF. In inflamed bone, the overall increase in susceptibility in areas of fat metaplasia is at least partly due to increased fat content.

## 1 INTRODUCTION

The spondyloarthritides are a group of inflammatory diseases involving the spine, lower limb joints, and entheses.^1^ New bone formation is a key feature of spondyloarthritis and causes spinal fusion, which contributes to pain, morbidity and disability. Conversely, spondyloarthritis patients may also suffer from bone loss in the form of osteoporosis,^2^ which contributes to increased fracture risk. Both disease processes cause alterations in bone mineral density (BMD), but this tissue property is difficult to measure using the conventional T_1_‐weighted and T_2_‐weighted short tau inversion recovery (STIR) spin echo images that are widely used in clinical practice.^3^, ^4^, ^5^, ^6^ Therefore, there is a clinical need for a quantitative, MRI‐based method for measuring BMD, and enable monitoring of new bone formation and bone loss in spondyloarthritis. An MRI‐based measure of BMD could also be useful for drug development, as there are several emerging therapies designed to inhibit bone formation in spondyloarthritis which currently lack a corresponding biomarker.^7^

Previously, Bray et al have proposed R_2_* as a quantitative biomarker of trabecular BMD as the diamagnetic nature of bony trabeculae is expected to increase the rate of signal decay.^8^ They found a positive correlation between BMD and R_2_* in a fat‐water‐bone phantom (a mixture of peanut oil, agar solution, and granules of bovine bone matrix), and also significantly reduced R_2_* in areas of fat metaplasia (an area with increased bone marrow fat content occurring in regions of previous inflammation)^9^ in patients with spondyloarthritis. However, R_2_* measurements are also influenced by variations in fat content, and the relationship between fat fraction (FF) measurements and R_2_* is complicated. This complexity arises because fat contributes to dephasing both within the voxel, because of the multipeak fat spectrum,^10^, ^11^ and in adjacent voxels, due to field inhomogeneities induced by magnetic susceptibility differences between water‐based and more paramagnetic fatty tissue.^12^, ^13^, ^14^ Furthermore, R_2_* measurements cannot differentiate between para‐ and diamagnetic structures.^15^

Recently, QSM^15^, ^16^, ^17^ has been investigated as an alternative method for quantifying BMD, with promising initial results.^18^, ^19^ Dimov et al showed that susceptibility values were closely correlated with CT measurements of BMD in a porcine hoof, and were able to generate susceptibility maps in which cortical bone was homogenous and diamagnetic, as expected from theory.^18^ However, susceptibility mapping is challenging in the presence of varying fat content, which is a characteristic feature of bone marrow inflammation in spondyloarthritis.^8^ Similarly to R_2_* measurements, susceptibility estimates can be confounded by variations in fat content, which contribute to dephasing both within the voxel (due to chemical shift) and in adjacent voxels (due to field inhomogeneities arising from the higher susceptibility of fat relative to water‐based tissue)^12^, ^13^, ^14^

In this study, we investigated the feasibility of QSM in inflamed bone marrow. The described QSM method was designed to correct for the effect of chemical shift. We also attempted to separate the fat contribution to total susceptibility so that “fat‐corrected” susceptibility measurements could be calculated. We evaluated the relationship between susceptibility, FF, and BMD in dedicated phantoms containing fat, water, and trabecular bone. Furthermore, we evaluated the differences in susceptibility between areas of normal marrow, edema, and fat metaplasia in patients with spondyloarthritis.

## 2 METHODS

This study received ethical approval from the Queen Square Research Ethics Committee, London, United Kingdom (Research Ethics Committee reference 15/LO/1475). All patients gave written informed consent before study entry.

### 2.1 Fat‐water‐bone phantom

**Figure 1:**
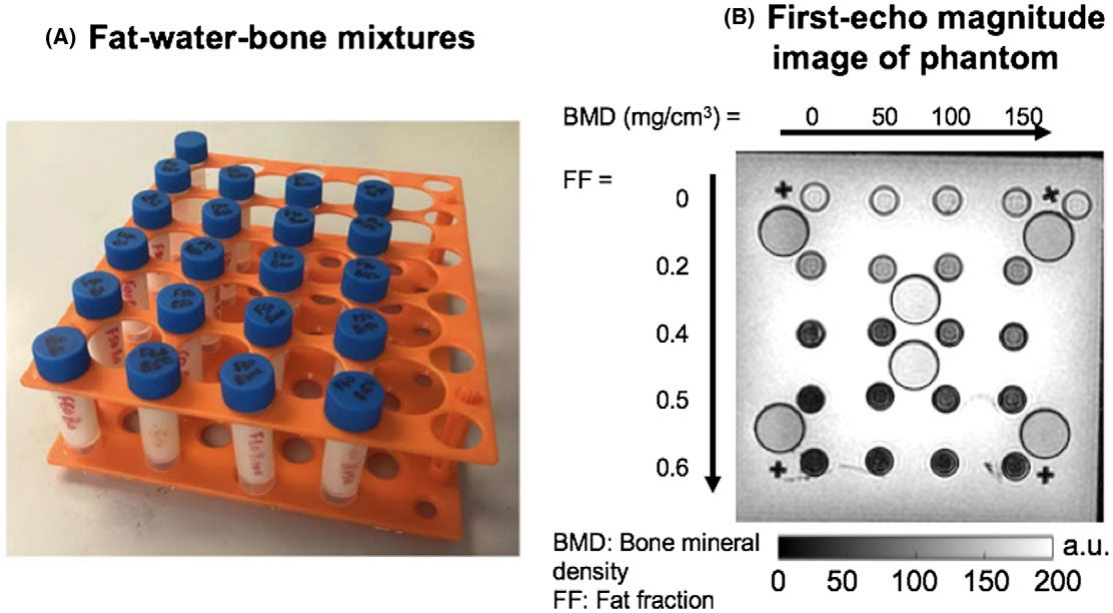
A,B, Fat‐water‐bone phantom. Fat fraction (FF) measurements vary by row, whereas bone mineral density (BMD) measurements vary by column. The phantoms were immersed in distilled water for scanning. The larger tubes shown in (A), interspersed between the columns of the phantom, contain fat‐water mixtures that were used as a visual check on the quality of fat‐water decomposition (i.e., to exclude fat‐water swaps) while optimizing the acquisition

We investigated the effect of FF and BMD on the calculated susceptibility using a fat‐water‐bone phantom (Figure 1) consisting of varying concentrations of peanut oil, water and decellularized bovine trabecular bone matrix, as previously described.^8^ This phantom consists of twenty 5‐mL scintillation vials with FF measurements varying by row and BMD measurements varying by column (Figure 1B), with the range of FF and BMD values (0‐60% and 0‐150 mg/cm^3^, respectively) designed to cover the range of values expected in both normal marrow and disease states.^8^, ^20^ As described previously, FF values in the phantom are calculated by volume and can be regarded as “reference” FF values rather than true proton density FF (PDFF) measurements, although the two parameters are expected to be very similar.^8^ The phantom was immersed in distilled water (without doping) for scanning.

### 2.2 Fat‐water phantom

A new fat‐water phantom was also created to examine the relationship between susceptibility and fat fraction over the full FF range (0‐100%). Eleven 50‐mL centrifuge tubes were filled with mixtures of water and lard (rather than peanut oil as lard enabled us to create stable, solid phantoms which did not separate at high FF), using sodium dodecyl sulfate as a surfactant, with FF values varying from 0% to 100%, in 10%‐increments. The final sodium dodecyl sulfate concentration in each phantom was 28 mM. Although this phantom allowed us to investigate the susceptibility at higher FF values, the dispersion of lard in water was poorer than that of peanut oil (in the fat‐water‐bone phantom) leading to visible clumping in the tubes with FF 50‐80%. Therefore, R_2_* values were not measured in this phantom. Again, this phantom was immersed in distilled water for scanning.

### 2.3 Patients and volunteers

This study was performed using data previously acquired by Bray et al^8^ in 18 patients (aged 12 to 30 years) diagnosed with or suspected of having spondyloarthritis. Patients with suspected spondyloarthritis were treated as controls if the subsequent clinical MRI scan and clinical assessment were found to be normal (*n* = 10).

### 2.4 Data acquisition

MRI magnitude and phase images of the fat‐water‐bone phantom and the subjects were acquired by Bray et al^8^ at 3T (Ingenia, Philips Healthcare, NL) using a 3D spoiled gradient‐echo pulse sequence with monopolar readout gradients, with integrated posterior and anterior surface coils (each with 16 channels). Images of the phantom were acquired coronally, with the following parameters: field of view = 30 × 30 × 80 cm^3^, resolution = 0.94 × 0.94 × 1.5 mm^3^, TE_1_ = 1.233 ms, ΔTE = 1.951 ms, 6 echoes, TR = 23 ms, flip angle = 3°, bandwidth 1159 Hz/pixel. Images of the patients and volunteers consisted of tilted coronal slices through the sacroiliac joints (parallel to the long axis of the sacrum), field of view = 50 × 50 × 80 cm^3^, resolution = 1.56 × 1.56 × 2 mm^3^, TE_1_ = 1.17 ms, ΔTE = 1.6 ms, 6 echoes, TR = 25 ms, flip angle = 3°, bandwidth 1894 Hz/pixel. Phantoms and subjects were also imaged using a similar vendor‐supplied gradient‐echo sequence with bipolar readout gradients (Philips, mDixon Quant, Philips Healthcare, Andover, MA),^10^ which provided PDFF and R_2_* maps with the same matrix size and field of view as the raw complex data. The mDixon Quant algorithm assumes a 10‐peak model of human adipose tissue and a single R_2_* decay term, as previously described.^10^, ^11^ Multiecho images of the new fat‐water phantom were acquired with the same sequence used for scanning the fat‐water‐bone phantom described above.

Subjects also underwent a standard clinical MRI scan on a 1.5T system (Avanto, Siemens, DE) with angled coronal (tilted at the same angle as the gradient‐echo images) T_1_‐ and T_2_‐weighted STIR sequences.^21^ These images were used only as landmarks for the manual segmentation of normal bone marrow, bone marrow edema, and fat metaplasia by an experienced radiology registrar (T.J.P.B.). Susceptibility mapping was only applied to the images acquired at 3T.

### 2.5 Susceptibility mapping (QSM) pipeline

Susceptibility maps were obtained from all multiecho images^22^ using the following, optimized QSM pipeline: (1) Three‐point Dixon method^23^ to estimate a field map without fat‐water chemical shift effects, (2) Laplacian phase unwrapping^24^ to remove temporal and spatial phase aliasing, (3) Projection onto dipole fields^25^ to remove background fields, and (4) direct k‐space inversion using Tikhonov regularization^26^, ^27^, ^28^ to calculate the susceptibility maps.

The three‐point Dixon method (step 1) requires only three equally spaced echoes. We used the first, third, and fifth echoes of both the phantom and subject images, as these consistently provided images with the fewest fat‐water swapping artifacts by visual inspection. All images were zero‐padded to a matrix size of 512 × 512 × 128 before steps 2 and 4 to avoid errors introduced by the application of direct and inverse Fourier transforms in these methods. The tilt of the coronal slices was accounted for by defining the dipole kernel to be parallel to the real direction of the main magnetic field in steps 3 and 4. The Tikhonov regularization parameter was set to α = 0.05 in step 4 based on the optimized value in Langkammer et al.^26^

The background field removal (step 3) requires a binary tissue mask. Initial masks were obtained in each case by thresholding the inverse noise map calculated from the multiecho magnitude images^27^, ^29^ to exclude high‐noise voxels that could introduce streaking into the susceptibility maps. In the phantoms, artifact‐inducing structures (i.e., the plastic struts of the vial holders) were manually segmented in the first‐echo magnitude images using ITK‐SNAP^30^, ^31^ and also excluded from the masks. In the patient and volunteer images, bony voxels were often excluded due to their high noise levels. However, this study aimed to calculate susceptibility maps in bone marrow so some of these noisy bony voxels were of interest. We could have adjusted the threshold for the masking step to include these bony areas in the tissue mask, but then other noisy, artifact‐inducing voxels (e.g., around the tissue/air interfaces in bowel) would be included as well. Therefore, we used the original threshold and added the excluded bone to the tissue mask later.

These bony voxels were identified in all subjects using the following scheme: (1) Bones were manually segmented (by A.K.) in the first‐echo magnitude image of one of the healthy volunteers (subject 1) in ITK‐SNAP.^30^, ^31^ (2) All scanner‐provided water images were thresholded so that values in regions with low water signal were set to zero. (3) The thresholded water image of subject 1 was nonrigidly registered to all other thresholded water images using the NiftyReg software^32^ with the weight of the bending energy term increased to 0.01 and a final grid size of 7 voxels. (4) Bones were segmented in the rest of the images by applying the resulting transformations to the manually segmented bone region of subject 1. This process provided suitable segmentations in all subjects. We used the thresholded water images here, because the shape and size of subcutaneous fat largely varied across subjects whereas the water images generally looked similar and, therefore, provided more accurate registrations around bony structures. Additionally, the edges of the patient and volunteer tissue masks were eroded by 5 voxels in each slice to further improve the quality of the susceptibility maps.

Because QSM calculates the average susceptibility of the substances (in other words the volume susceptibility)^33^, ^34^ within each voxel, it is expected to have a linear relationship with both fat and bone content (i.e., PDFF and BMD). Therefore, we propose a procedure to estimate BMD‐induced susceptibility maps: (1) PDFF and susceptibility maps were measured and calculated. (2) Linear regression was performed between susceptibility and PDFF in voxels without bony trabeculae. (3) The regression parameters and the PDFF map were then used to estimate the contribution of fat to susceptibility in every voxel. (4) The contribution of fat was subtracted from the total susceptibility map resulting in a susceptibility map that is expected to be proportional to BMD assuming that no other para‐ or diamagnetic components are present. We performed this procedure in all volunteer and patient susceptibility maps. Step 2 was carried out in a rectangular region, including both water‐based tissue (gluteal muscle) and subcutaneous fat, manually selected in the middle slice in each subject.

To compare the contributions of BMD with susceptibility and R_2_*, a similar procedure was performed for the scanner‐provided R_2_* maps. To model the effects of PDFF on the measured R_2_*,^8^ we adopted an empirical quadratic fit (instead of the aforementioned linear relationship) in step 2 that provided better fits.

### 2.6 Statistical analysis

For both phantoms, circular regions of interest (ROIs) were manually drawn (by A.K.) on the first‐echo magnitude images in eight consecutive slices near the middle of the acquired volumes using ITK‐SNAP.^30^, ^31^ Mean susceptibilities and R_2_* values were calculated in all ROIs. The 2D linear functions were fitted to the measured susceptibility and R_2_* values as functions of known FF and BMD values in the fat‐water‐bone phantom. Linear regression was performed between measured susceptibilities and known FF values in the fat‐water phantom.

For the patients and healthy controls, areas of normal bone marrow, bone marrow edema, and fat metaplasia were manually segmented on the first‐echo magnitude images using landmarks from the T_2_‐weighted STIR and T_1_‐weighted images by an experienced radiology resident (T.J.P.B.) as described in Bray et al.^8^ ROIs that were very close to fat‐water chemical shift artifacts in the susceptibility maps were excluded from the analysis. This included all ROIs from two subjects (8 ROIs of fat metaplasia and 4 ROIs of normal bone marrow), and two additional ROIs from a third subject (of 7 ROIs of fat metaplasia). Mean susceptibilities and R_2_* values were calculated in the rest of the segmented ROIs both before and after removing the contributions of fat as described above (i.e., the same ROIs were used in all four cases). Susceptibility values were referenced to the mean susceptibility within the tissue mask for each subject (this mean susceptibility varied between −0.01 ppm and 0.01 ppm). Multilevel mixed‐effects linear regression was used (in MATLAB R2015a) to determine whether there were significant differences in susceptibilities and R_2_* values measured in normal bone marrow, edema, and fat metaplasia. This test accounts for repeated observations in individual patients.

## 3 RESULTS

### 3.1 Phantoms

**Figure 2:**
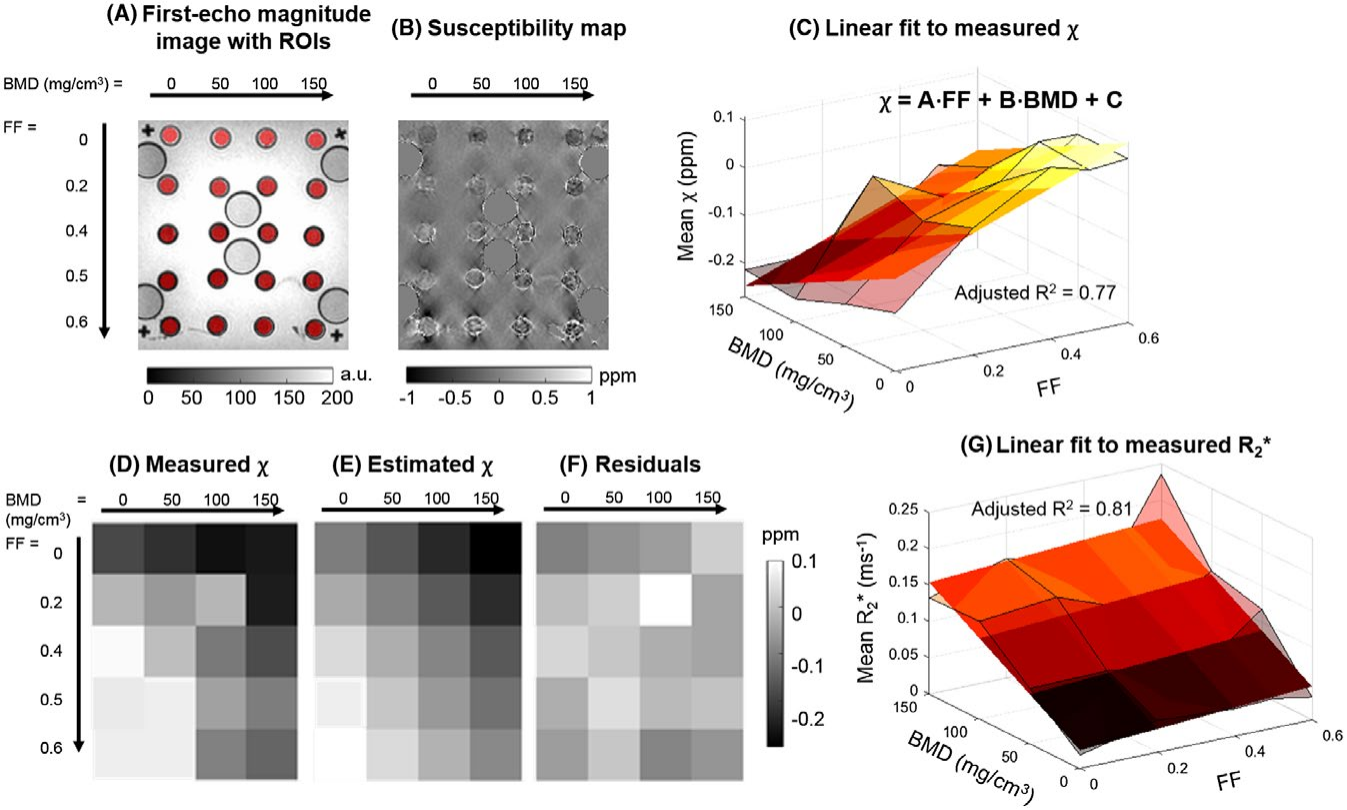
Results from the fat‐water‐bone phantom. First‐echo magnitude image and susceptibility map are shown in (A) and (B), respectively. The manually drawn circular regions of interest (ROIs) are highlighted in red (A). Results of the 2D linear fit between bone mineral density (BMD) and fat fraction (FF) values and susceptibility are shown in (C‐F). In (C), the transparent surface corresponds to measured values, while the opaque plane is the fitting 2D linear function. The same is shown for R_2_* in (G). The fitted model parameters are shown in Supporting Information Table S1a, b

**Figure 3:**
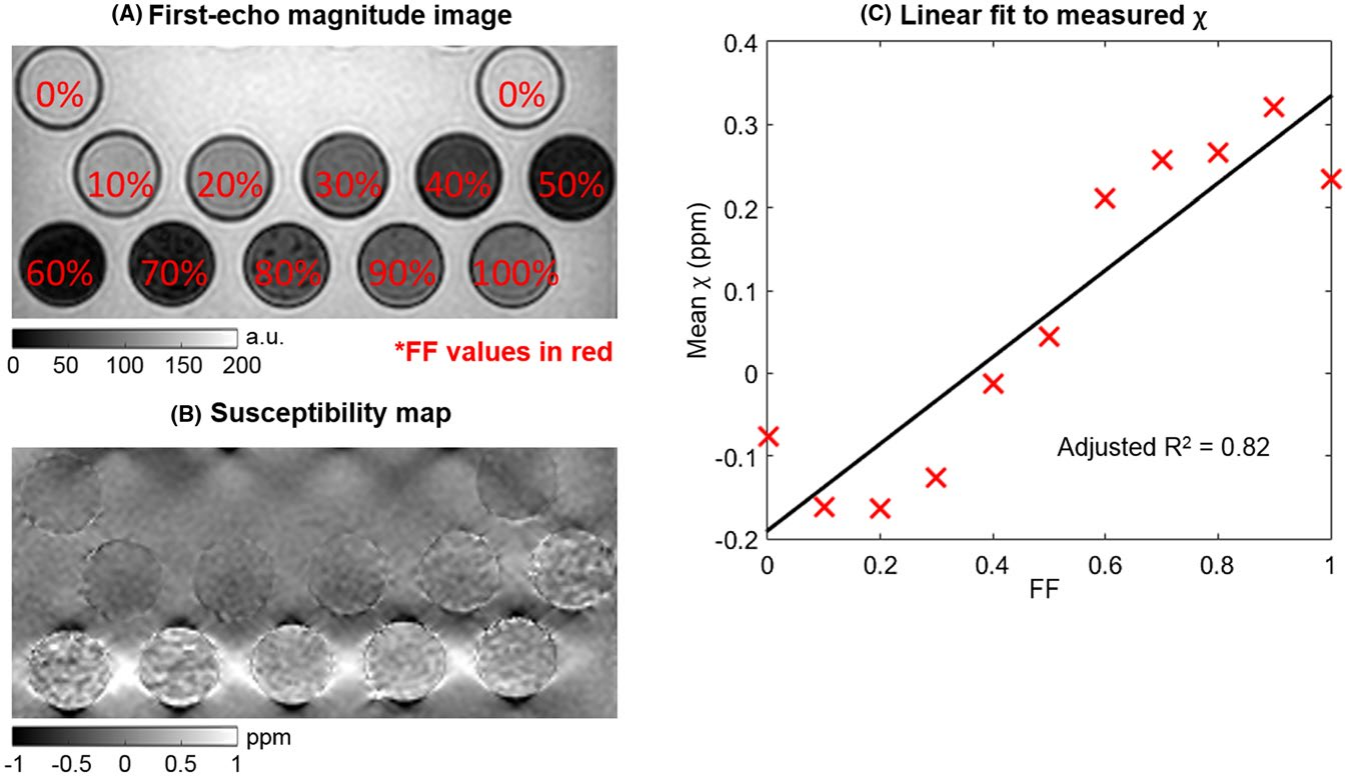
Results from the fat‐water phantom. First‐echo magnitude image and susceptibility map are shown in (A) and (B), respectively. The linear fit between fat fraction (FF) and susceptibility values is shown in (C). The fitted model parameters are shown in Supporting Information Table S1c

Images from the fat‐water‐bone phantom are shown in Figure 2A,B. Susceptibility measurements were positively related to FF values and negatively related to BMD (Figure 2C, Supporting Information Table S1a), with the 2D linear model providing an accurate description of the acquired data (adjusted R^2^ = 0.77; Figure 2C‐F). All coefficients were significant (i.e., *P* < 0.01, Supporting Information Table S1a). Similarly, in the lard‐water phantom covering the full range of FF values (Figure 3), there was an approximately linear relationship (adjusted R^2^ = 0.82) between FF and susceptibility (Figure 3C, Supporting Information Table S1b) even toward high FF values. Figure 2G shows the 2D linear model fitted to the R_2_* values. In this case, despite the high adjusted R^2^ value (0.81), only the BMD slope was significant (Supporting Information Table S1b).

### 3.2 Patients and volunteers

**Figure 4:**
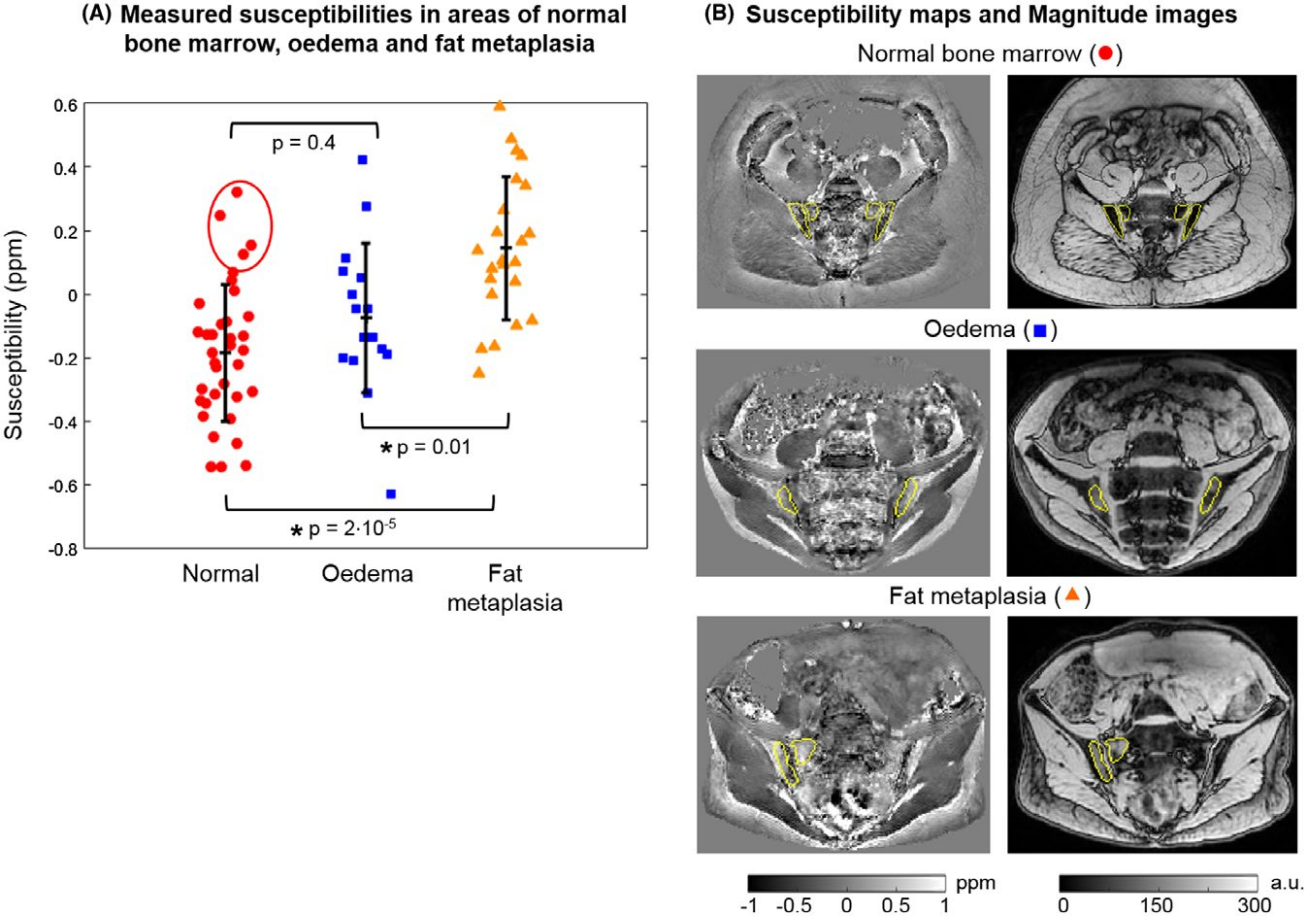
Susceptibility maps in patients. Measured mean susceptibilities in areas of normal marrow, edema, and fat metaplasia are shown in (A). *P*‐values were calculated for each pair and the asterisks indicate statistical significance. The four highest susceptibility values in normal bone marrow (red circle) were measured in the same subject. Susceptibility maps and magnitude images in example subjects are shown in (B)

**Figure 5:**
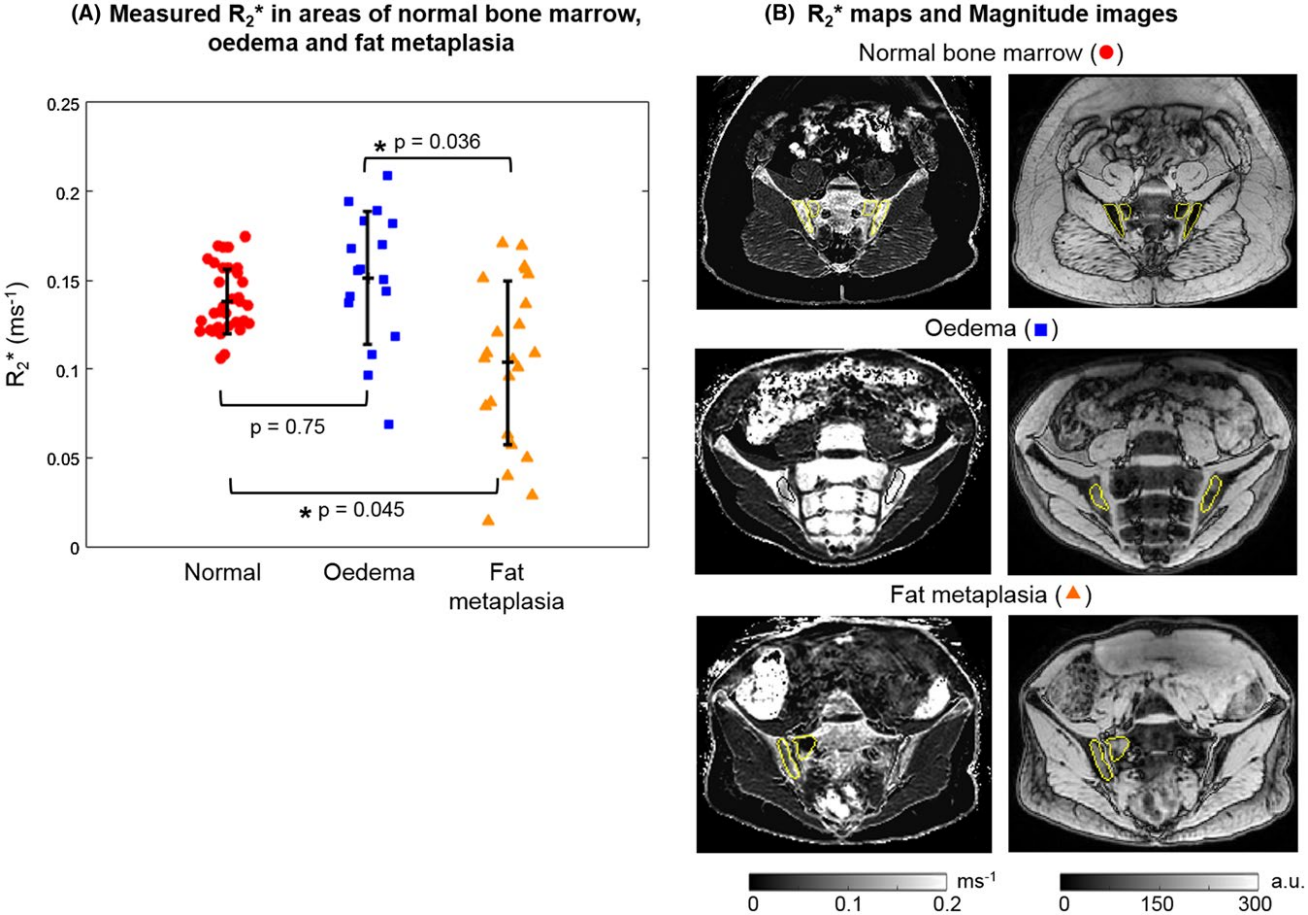
R_2_* maps in patients. Mean R_2_* measurements in areas of normal marrow, edema, and fat metaplasia are shown in (A). *P*‐values were calculated for each pair and the asterisks indicate statistical significance. R_2_* maps and magnitude images in example subjects are shown in (B)

Susceptibility values were significantly increased in areas of fat metaplasia compared with normal marrow (Figure 4A). R_2_* measurements were also significantly reduced in areas of fat metaplasia compared with normal marrow (Figure 5A), in line with previous results.^8^ There were no significant differences in susceptibility or R_2_* between normal bone marrow and areas of edema. However, susceptibility values were significantly lower, and R2* values significantly higher, in areas of edema compared with areas of fat metaplasia.

**Figure 6:**
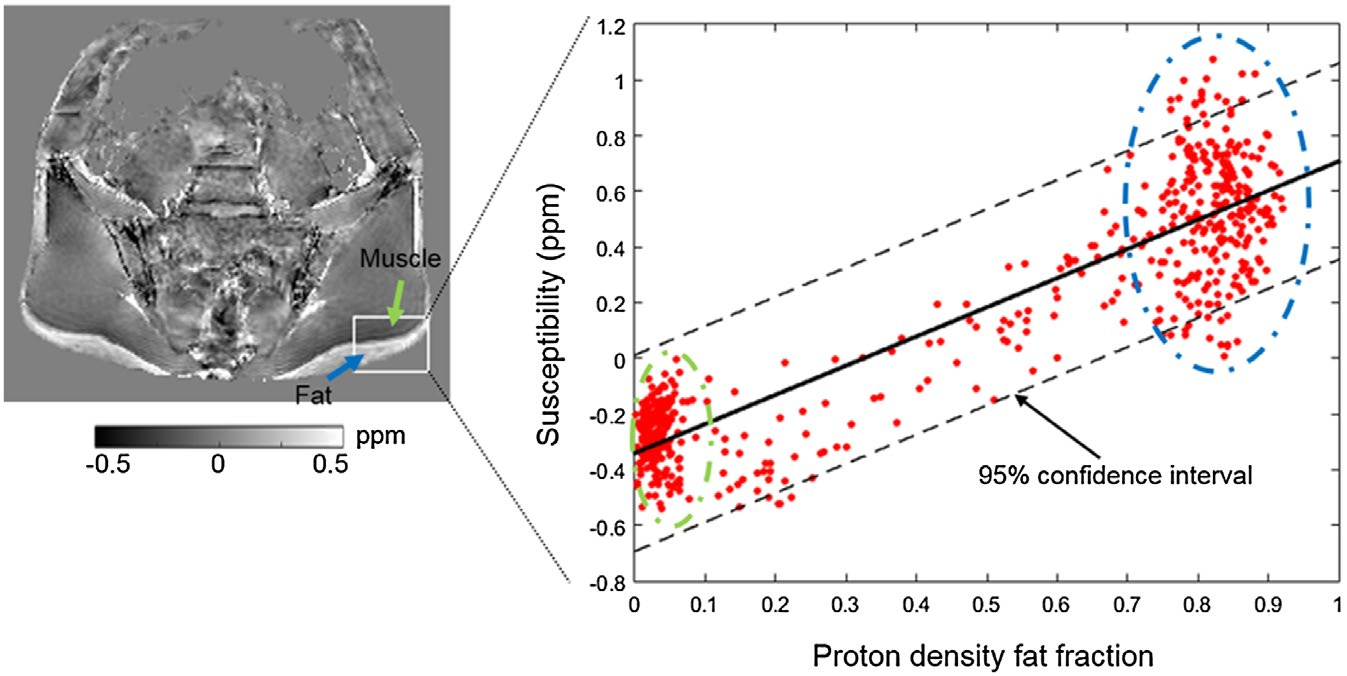
Linear regression between proton density fat fraction and susceptibility values in a single, representative subject. A rectangular ROI including fat and muscle was manually placed on the middle slice of the susceptibility map. Each point in the scatter plot corresponds to one voxel within this rectangular region (excluding voxels outside the subject). The blue arrow and dotted circle indicate subcutaneous fat and the corresponding points in the scatter plot. The green arrow and dotted circle indicate muscle and the corresponding points in the scatter plot. Results from the regression analysis for each subject are shown in Figure 8

**Figure 7:**
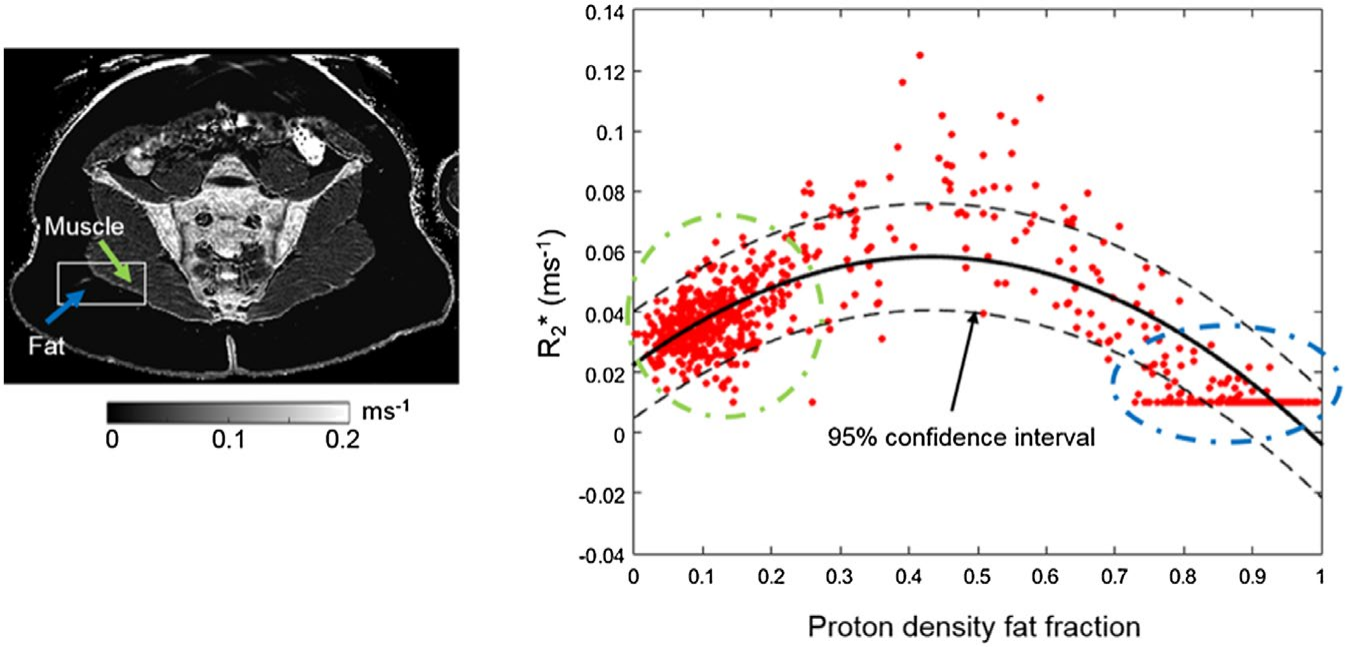
Nonlinear regression between PDFF and R_2_* values using a quadratic model. The R_2_* map and the regression of a representative subject are shown. Each point in the scatter plot corresponds to one voxel within this rectangular region (excluding voxels outside the subject). The blue arrow and dotted circle indicate subcutaneous fat and the corresponding points in the scatter plot. The green arrow and dotted circle indicate muscle and the corresponding points in the scatter plot. Results from the regression analysis for each subject are shown in Figure 8. Note that the straight line of points at the right lower corner of the plot arises due to the lower bound applied to R_2_* estimates by the mDixon Quant fitting algorithm

**Figure 8:**
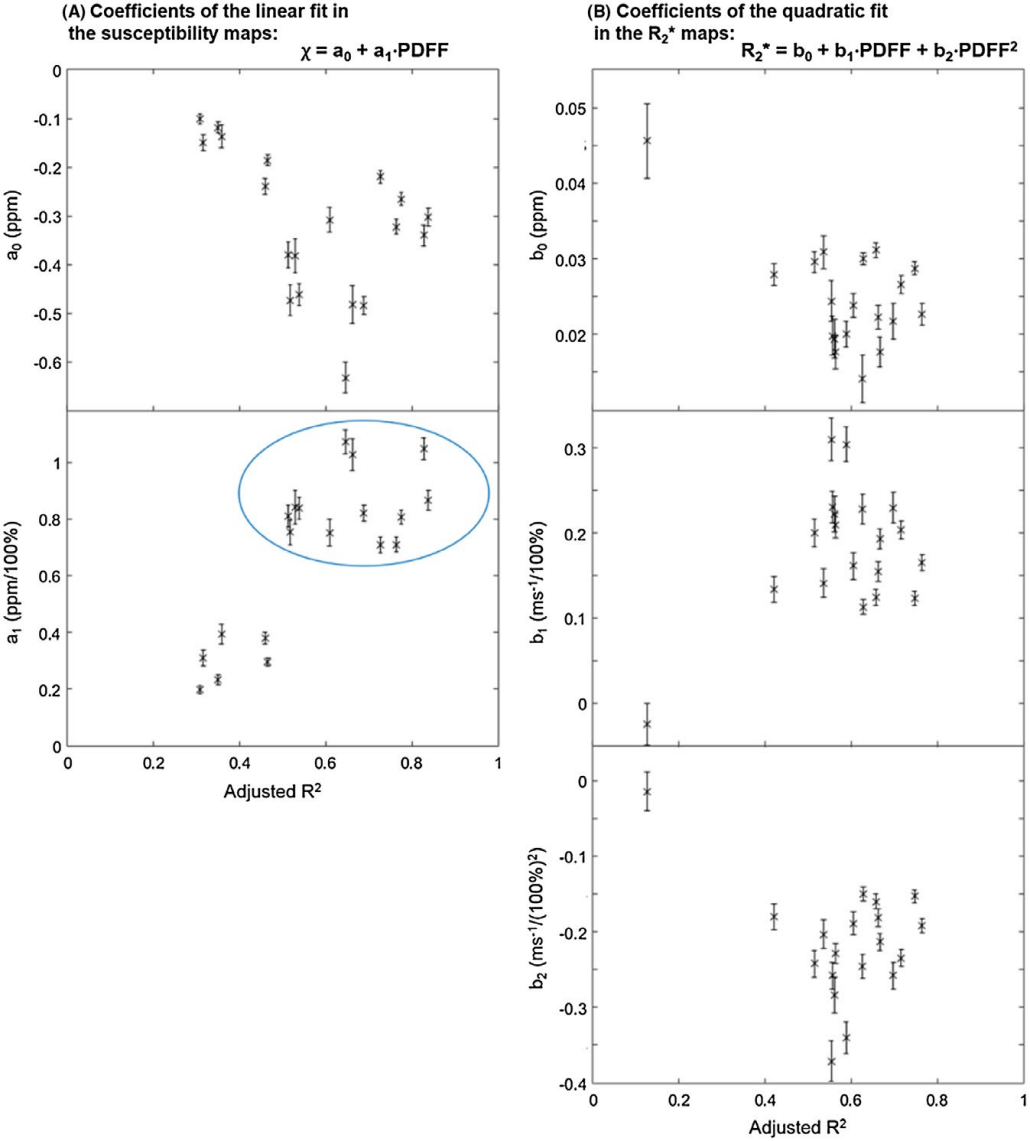
Coefficients of the linear and quadratic fits between proton density fat fraction and susceptibility (A) or R_2_* (B), respectively. The fitting function is displayed in the top right corner of both subplots. In all 5 scatter plots, each point corresponds to 1 subject. Coefficients are shown as a function of the adjusted R^2^ of each fit. The error bars indicate the 95% confidence interval of each coefficient. The slope of the linear regression (**a_1_**) seems to be consistent in instances where the adjusted R^2^ was high (>0.5, see blue circle). All other parameters had large variations across subjects

PDFF and susceptibility values within a single, rectangular ROI (overlaid on the susceptibility map) incorporating both muscle and subcutaneous fat, in addition to the results of the linear regression analysis, are shown for a single subject in Figure 6. Similarly, Figure 7 shows R_2_* and PDFF values within the manually selected ROI (overlaid on the R_2_* map), and the results of the nonlinear regression analysis assuming a quadratic relationship. In both Figures 6 and 7, the majority of voxels are either almost entirely water based (green arrow and dotted circle) or fat based (blue arrow and dotted circle). Note that the straight line observed at the upper end of the PDFF range in Figure 7 arises due to a lower bound for R_2_* estimates (0.01 ms^−1^) used by the mDixon Quant algorithm. Model parameters from the linear and quadratic fits between PDFF and susceptibility, and PDFF and R_2_*, respectively, are shown in Figure 8. The coefficients of the quadratic fit (Figure 8B) were highly variable across subjects. While the intercept of the linear fit to susceptibility values also showed large variations across subjects, the susceptibility‐PDFF slope was somewhat consistent for regressions with high adjusted R^2^ measures (Figure 8A, blue circle).

**Figure 9:**
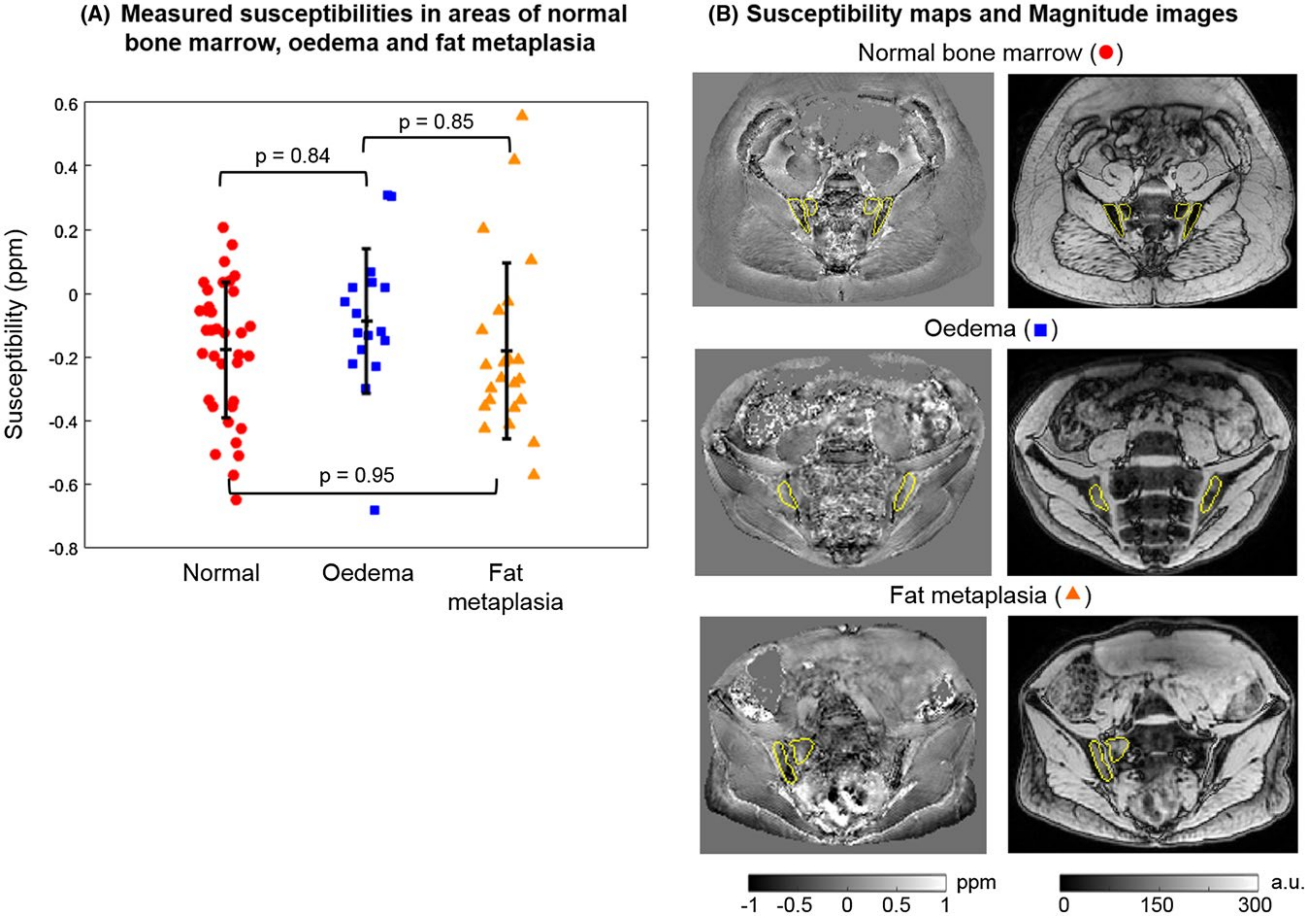
Susceptibility maps in patients after removing the fat contribution. Fat‐corrected susceptibility measurements in areas of normal marrow, edema and fat metaplasia are shown in (A). *P*‐values are calculated for each pair. Susceptibility maps and magnitude images in example subjects are shown in (B)

**Figure 10:**
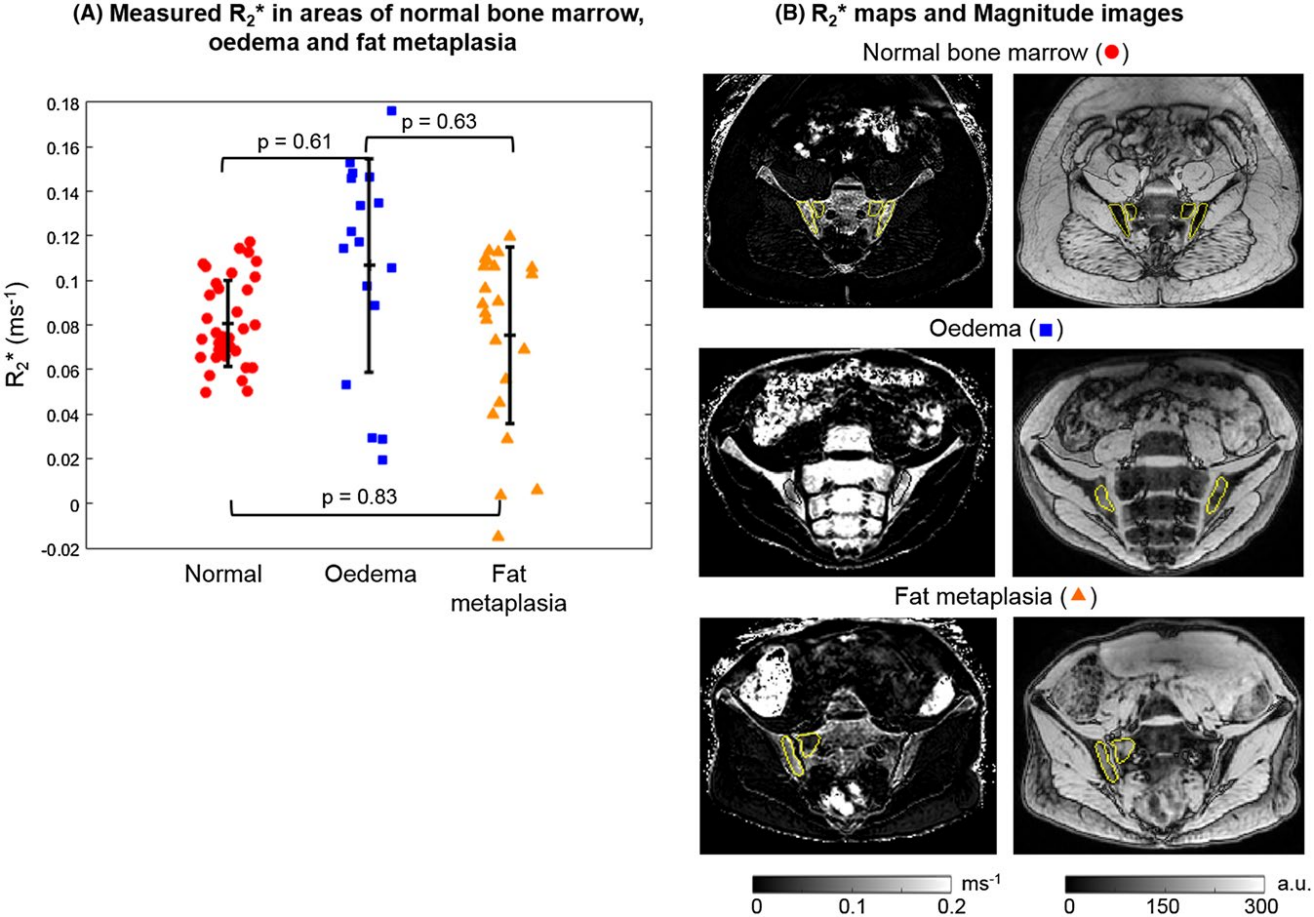
R_2_* maps in patients after removing the fat contribution. Fat‐corrected R_2_* measurements in areas of normal marrow, edema, and fat metaplasia are shown in (A). *P*‐values were calculated for each pair. R_2_* maps and magnitude images in example subjects are shown in (B)

Susceptibility values and R_2_* measurements after removing the fat contribution are shown in Figures 9 and 10, respectively. There were no significant differences between normal bone marrow and fat metaplasia in either susceptibility or R_2_* maps after performing the adjustment for fat content.

## 4 DISCUSSION

In spondyloarthritis, new bone formation and bone destruction contribute to spinal ankylosis and osteoporosis, respectively, and are key contributors to morbidity. However, these processes cannot be quantified using conventional spin echo sequences. In this study, we sought to characterize the relationship between BMD, FF, and susceptibility measurements in inflamed bone marrow, using both phantom and in vivo studies with the aim of investigating susceptibility as a potential biomarker of these processes.

In the fat‐water‐bone phantom, we observed linear relationships between FF and susceptibility and between BMD and susceptibility. The linear relationship between FF and susceptibility was also observed in a separate, lard‐based fat‐water phantom covering the full range of FF measurements, although there was a slight nonlinear variation which may be related to difficulties in manufacturing a homogenous lard‐water phantom. In accordance with previous studies,^12^, ^13^, ^14^, ^18^ our data indicated positive (paramagnetic) susceptibility values for fat, and negative (diamagnetic) susceptibility values for bone. Our results confirm the feasibility of measuring BMD in the bone marrow, and suggest that the contribution of fat to the total susceptibility measurement can be modeled using a simple linear relationship.

Importantly, the results of our phantom study were used to inform the analysis of the in vivo results, to enable us to estimate the contribution of the fat to the total susceptibility measurement. Using per‐patient linear regression analysis in voxels of subcutaneous fat and muscle, we were able to remove the fat contribution to susceptibility measurements in areas of fat metaplasia, edema, and normal marrow, and thereby interrogate the source of susceptibility differences between these regions. Strikingly, we found that susceptibility measurements were significantly increased in areas of fat metaplasia compared with normal marrow, but that this difference was abolished after removal of the fat contribution. This suggests that the contribution of fat content to overall susceptibility is likely to be substantial, and highlights the importance of accounting for the contribution of fat when performing QSM in bone marrow.

Similarly, although there was a significant reduction in R_2_* in areas of fat metaplasia compared with normal marrow, no significant difference was observed in fat‐corrected R_2_* measurements. This result suggests that the previously‐reported reduction in R_2_*^8^ in areas of fat metaplasia may actually be a secondary effect of varying fat content: as fat fraction increases from around 50% (normal bone) to approximately 70‐90% (fat metaplasia),^8^ the susceptibility distribution inside the voxel becomes more homogenous, and the relaxation rate R_2_* reduces accordingly. This suggestion is in keeping with previous results in muscle, which suggest that R_2_* measurements are highest at intermediate PDFF values, and are at their lowest at the extremes of the PDFF range (i.e., close to 0% and close to 100%).^35^ In contrast, the linear relationship of susceptibility with FF suggests that susceptibility measurements are less affected by the microscopic (sub‐voxel) susceptibility distribution, because the susceptibility calculation largely relies on larger‐scale phase differences outside the voxel. This is potentially a significant advantage of QSM over R_2_* mapping.

Overall, the results of our study highlight that lipids contribute substantially to both susceptibility and R_2_* estimates in trabecular bone, and can at least partly account for the differences in susceptibility between regions. Accounting for the fat contribution to susceptibility is likely to be essential when imaging trabecular bone in general. If the fat contribution is not separated, changes in susceptibility/R_2_* might be incorrectly attributed to changes in BMD, or other factors.

In this study, we used the individual regression parameters in Figure 8 for each subject to remove the contributions of fat from susceptibility and R_2_* maps. For the R_2_* maps, the quadratic model is a heuristic approximation of the observed shape of the PDFF‐ R_2_* relationship. Therefore, the coefficients vary greatly across subjects (Figure 8B), and the correction is expected to be more accurate if individual fitting parameters are used.

In QSM, the susceptibility of a tissue can only be measured relative to the susceptibility of surrounding tissues, and there is expected to be variation in this susceptibility offset across subjects. This could explain the variability of a_0_ in Figure 8A. Though referencing is used to enable comparison of susceptibility across scans, here we used the mean susceptibility within the tissue mask as a reference which might not be ideal. Future studies could explore other potential reference tissues in the pelvic area similarly to what has been done in the brain.^36^ This could result in more consistent a_0_ values across subjects. The estimated slope (a_1_ in Figure 8A) seems to be consistent across subjects for regressions with high adjusted R^2^ (Figure 8A, blue circle). This is encouraging as the composition of subcutaneous fat and, therefore, the relationship between FF and susceptibility is expected to be similar across subjects. In a few cases (where the adjusted R^2^ was lower), the fitted slopes as well as the correlation between susceptibility and FF (not shown) were substantially lower. This might be due to susceptibility errors on the boundary between fat‐ and water‐based tissues introduced by the large susceptibility gradient.

Selecting two separate areas (one in subcutaneous fat and one in muscle) could be a way of overcoming this problem in the future; however, here we needed to include the voxels on the boundary (where FF values are between 0.3 and 0.7) to be able to appreciate the relationships between FF, and susceptibility or R_2_* (Figures 6 and 7). It could be interesting to explore if a single slope value can be used to robustly remove fat contributions from susceptibility maps. In any case, using individual regression parameters was more appropriate here as the same approach was implemented for the R_2_* maps. Of interest, the estimated slope values were different in subjects than in the fat‐water phantoms (and also varied slightly between the two phantoms), which might be due to differences in susceptibility between different lipids or, in patients, to susceptibility contributions from nonlipid molecules in muscle and/or adipose tissue. Further work is required to investigate this issue.

It is not yet known how bone content or structure changes in areas of edema and fat metaplasia. In the patients with spondyloarthritis investigated here, the fact that we did not find a significant difference in fat‐corrected susceptibility in areas of fat metaplasia argues against a significant change in BMD in these areas. However, it is also possible that we have simply failed to detect this change due to technical limitations arising from the acquisition protocol and/or susceptibility mapping pipeline such as the removal of chemical‐shift‐induced errors, as discussed in the next paragraph. Also, note that the four highest susceptibility values in normal marrow (Figure 4A, red circle) were measured in the same subject, so these unrealistically high values could be due to a processing issue in this subject. We did not find a significant difference in susceptibility between areas of edema and normal bone marrow. On theoretical grounds, we would have expected a reduction in susceptibility (before fat correction) in areas of edema due to increased water fraction, which would have been expected to disappear after fat correction. However, the changes in fat fraction in areas of edema are smaller than those observed in fat metaplasia (compared with normal marrow),^8^ which may have prevented detection of this effect.

A limitation of this study is that the fat‐water decomposition step in the QSM pipeline, aiming to eliminate chemical shift effects, suffered from fat‐water swaps in some subjects, which may have contributed to inaccuracies in the calculated susceptibilities. Swaps may be introduced during the region‐growing stage of the fat‐water decomposition (Dixon) method,^37^ possibly due to errors or noise in the measured phase data. Empirically, we found that the three‐point Dixon method used here was the most robust of the available options in the ISMRM fat‐water toolbox,^37^ although even this did not perform perfectly in all cases. Better results might be achieved by using alternative algorithms for fat‐water decomposition. One option is to use manufacturers’ own algorithms for fat‐water decomposition (and to generate field maps), but this comes at the cost of reduced flexibility and makes it more difficult to translate the approach to other platforms. Another possibility is using in‐phase echo timing to remove most of the chemical shift phase contributions,^38^, ^39^ while also acquiring opposed‐phase (or partially opposed‐phase) images to calculate PDFF maps.

One of the most crucial features of susceptibility mapping is the generation of the tissue mask. Noisy voxels are prone to introduce far‐reaching streaking artifacts and errors into the susceptibility maps. In images of the sacroiliac joint, it is very important to properly exclude areas of bowel as the phase measured in these voxels is often corrupted by motion artifacts and suffers from low signal due to air in the bowel lumen. Therefore, thresholding the inverse noise map seems appropriate here for generating a suitable tissue mask. However, bony voxels are also expected to have low signal. The process described here, aiming to keep bony voxels while excluding bowel, was simple and provided reasonable tissue masks in most cases, but susceptibility accuracy could potentially be improved using more accurate, automated bone segmentation tools, for example, based on multiatlas information.^40^

There is a complex relationship between PDFF and R_2_* measurements, which means that changes in R_2_* in tissue are ambiguous. It might be possible to model this relationship using prior knowledge of fat and water susceptibility and the arrangement of fat and water in the tissue, but this is not trivial and introduces further sources of complexity. Using susceptibility as a marker of BMD has several advantages over R_2_*. Most importantly, the linear relationships observed in the fat‐water‐bone phantom enable fat‐correction to be performed very simply, and the fat‐corrected susceptibility measurements to be interpreted unambiguously. Future studies could correlate the measured bone marrow susceptibilities with gold‐standard, QCT‐based clinical BMD measures,^41^ however, this is subject to ethical constraints relating to the use of ionizing radiation, particularly in young patients.

## 5 CONCLUSIONS

Quantitative susceptibility measurements are linearly related to BMD and FF, and failure to remove the fat contribution to susceptibility measurements can potentially lead to errors in QSM‐based BMD quantification. We propose a method for removing this contribution using a linear fit to susceptibility as a function of FF in a region not containing bone. Comparison of data both with and without this correction suggest that increased fat content is the major contributor to the increase in susceptibility in areas of fat metaplasia relative to normal bone marrow.

## Supporting information


**TABLE S1** Fitted model parameters for the two phantoms. Result of the 2D linear fit between bone mineral density and fat fraction values and susceptibility in the fat‐ water‐bone phantom are shown in (A). The same for R2* is shown in (B). Results of the linear fit between fat fraction and susceptibility in the fat‐water phantom are shown in (C)
