# Supplementary material for: Association of bone mineral density and fat fraction with magnetic susceptibility in inflamed trabecular bone
Source: Magn Reson Med. 2019 Jan 7;81(5):3094–107. doi: 10.1002/mrm.27634 (PMC6492090; doi:10.1002/mrm.27634)
Supplement: Supplementary file 1 — TABLE S1 Fitted model parameters for the two phantoms. Result of the 2D linear fit between bone mineral density and fat fraction values and susceptibility in the fat‐ water‐bone phantom are shown in (A). The same for R2* is shown in (B). Results of the linear fit between fat fraction and susceptibility in the fat‐water phantom are shown in (C) [file MRM-81-3094-s001.pdf]

**a) Fat-Water-Bone Phantom (Susceptibility)**	Estimated value	Units	95% conf. int.	p-value
Fat fraction slope (A)	0.31	ppm/100%	(0.20, 0.42)	$2 \cdot 10^{-5}$
Bone mineral density slope (B)	-0.11	ppm/100$\frac{mg}{cm^3}$	(-0.16, -0.07)	$3 \cdot 10^{-5}$
Offset (C)	-0.08	ppm	(-0.13, -0.02)	$9 \cdot 10^{-3}$

**b) Fat-Water-Bone Phantom (R_2*)**	Estimated value	Units	95% conf. int.	p-value
Fat fraction slope (A)	0.036	ms^{-1}/100%	(-0.017, 0.089)	0.17
Bone mineral density slope (B)	$8.6 \cdot 10^{-4}$	ms^{-1}/100$\frac{mg}{cm^3}$	($6.6 \cdot 10^{-4}$, $10.7 \cdot 10^{-4}$)	$8 \cdot 10^{-8}$
Offset (C)	0.025	ms^{-1}	(-0.001, 0.051)	0.064

**c) Fat-Water Phantom (Susceptibility)**	Estimated value	Units	95% conf. int.	p-value
Fat fraction slope	0.52	ppm/100%	(0.35, 0.70)	$2 \cdot 10^{-4}$
Offset	-0.19	ppm	(-0.29, -0.09)	$5 \cdot 10^{-3}$

Supporting Information Table S1: Fitted model parameters for the two phantoms. Result of the 2D linear fit between bone mineral density and fat fraction values and susceptibility in the fat-water-bone phantom are shown in (a). The same for R_2* is shown in (b). Results of the linear fit between fat fraction and susceptibility in the fat-water phantom are shown in (c).
